# Genomic and Proteomic Analyses of Bacterial Communities of *Ixodes scapularis* Ticks from Broome County, New York

**DOI:** 10.3390/microorganisms13020258

**Published:** 2025-01-24

**Authors:** Michel Shamoon-Pour, Emily H. Canessa, John Macher, Amaan Fruitwala, Emma Draper, Benjamin Policriti, Matthew Chin, Matthew Nunez, Paul Puccio, Yuan Fang, Xin-Ru Wang, Yetrib Hathout

**Affiliations:** 1 First-year Research Immersion, Binghamton University, Binghamton, NY 13902, USA; 2 Department of Anthropology, Binghamton University, Binghamton, NY 13902, USA; jmacher1@binghamton.edu; 3 Tick-borne Disease Center, Binghamton University, Binghamton, NY 13902, USA; 4 Department of Pharmaceutical Sciences, School of Pharmacy and Pharmaceutical Sciences, Binghamton University, Binghamton, NY 13902, USAedraper1@binghamton.edu (E.D.);; 5 Department of Microbiology and Immunology, Upstate Medical University, Syracuse, NY 13210, USA; wangxin@upstate.edu

**Keywords:** *Ixodes scapularis*, Lyme disease, tick-borne diseases, microbiome, proteomics, *Borreliella burgdorferi*, *Rickettsia buchneri*, endosymbiont

## Abstract

The microbial communities of *Ixodes scapularis*, the primary vector of Lyme disease in North America, exhibit regional variations that may affect pathogen transmission and vector competence. We analyzed bacterial communities in *I. scapularis* ticks collected from Broome County, New York, using 16S rRNA gene sequencing (18 ticks) as well as mass spectrometry-based proteomics (36 ticks). According to the 16S rRNA analysis, the endosymbiont *Rickettsia buchneri* was the most abundant species, with significantly higher (*p* = 0.0011) abundance in females (54.76%) compared to males (31.15%). We detected *Borreliella burgdorferi* in 44.44% of ticks and *Anaplasma phagocytophilum* in two nymphs but in high relative abundances (12.73% and 46.46%). Male ticks exhibited higher bacterial diversity, although the community composition showed no significant clustering by sex or life stage. Co-occurrence analysis revealed negative associations between *R. buchneri* and *Pseudomonas* (*p* = 0.0245), but no associations with *B. burgdorferi*. Proteomic analysis identified 12 *R. buchneri*-specific proteins, additionally detecting the protozoan pathogen *Babesia microti* in 18.18% of females. These findings provide the first comprehensive characterization of *I. scapularis* microbiomes in the Southern Tier region of New York and suggest broader distribution of *R. buchneri* across tick life stages than previously recognized, with potential implications for pathogen transmission dynamics.

## 1. Introduction

*Ixodes scapularis*, commonly known as the blacklegged tick or deer tick, is an important vector of human pathogens in the United States, responsible for transmitting a wide array of tick-borne diseases (TBDs) [1,2]. Lyme disease, caused by the bacterium *Borreliella* (*Borrelia*) *burgdorferi* [3], is the most prevalent of these diseases, with approximately 500,000 cases reported annually [4]. The population density of *I. scapularis* has been rising in endemic areas, especially in the Northeastern and Mid-Atlantic USA. This increase is driven by several factors, particularly climate change, which is modifying the habitats and extending the geographic range of these ticks [5,6]. Other contributing factors include changes in land use, growing deer populations, particularly white-tailed deer (*Odocoileus virginianus*), and various ecological shifts [7,8,9,10]. As a result, *I. scapularis* ticks and the pathogens they carry are expanding into new regions of the USA and Canada, presenting a serious threat to public health [11,12].

Along with *B. burgdorferi*, *I. scapularis* may carry and transmit a range of less common human pathogens. In New York State, *I. scapularis* ticks have been shown to harbor at least four more tick-borne pathogens (TBPs) [13,14]: *Anaplasma phagocytophilum*, the bacterial agent of anaplasmosis [15], *Babesia microti*, a protozoan blood parasite [16], as well as less common *Borrelia miyamotoi* [17] and Powassan Virus [18]. *Ixodes scapularis* is also capable of transmitting other pathogens, including the bacteria causing ehrlichiosis (*Ehrlichia muris eauclairensis* and *Ehrlichia chaffeensis*) [19], as well as *Borreliella mayonii*, a Lyme disease-causing bacteria of the upper Midwestern USA [20]. Furthermore, co-infection of *I. scapularis* with tick-borne pathogens (TBPs) has been increasingly reported, and in rare cases, four pathogens have been detected in a single tick [21]. The increasing occurrence of TBP co-infections further complicates the diagnosis and treatment of TBDs [22]. 

The coexistence of TBPs with various other microorganisms within ticks is well documented [23,24]. These microbiota can affect the tick vector in multiple ways, including influencing nutritional adaptation, growth, reproduction, and defense against environmental stress [25,26]. Furthermore, variations in microbiomes, particularly of midgut and salivary glands, may affect *I. scapularis* physiology and the immune response, consequently altering the tick’s “vector competence”. For instance, it has been suggested that the presence of *Pseudomonas* spp., such as antibiotic-producing *P. fluorescens*, can limit the ability of *Ixodes* ticks to transmit Lyme disease by inhibiting *Borrelia* spp. [27,28,29]. *Rickettsia buchneri* is a key endosymbiont in the biology of *Ixodes scapularis*, providing essential nutrients such as B vitamins, which are crucial for the tick’s development and reproduction [27]. It enhances the tick’s defense against pathogens by producing antimicrobial compounds that suppress competing bacteria [30]. *Rickettsia buchneri* also contributes to the tick’s overall fitness by modulating the immune response and processes like autophagy [31], creating an environment that supports the persistence of tick-borne pathogens and impacting disease transmission dynamics. In addition to *R. buchneri*, associations between *B. burgdorferi* and a number of other bacteria [27,28,32,33] and viruses [34] of *I. scapularis* have also been proposed. Overall, there is significant potential for further research on tick microbiomes, which could provide deeper insights into the interactions between ticks, their microbiota, and the pathogens they transmit. Alongside 16S rRNA gene sequencing and other genetic methods for identifying microbial species in ticks [35], mass spectrometry-based proteomics offers a supplementary and more specific method for pathogen identification [36,37,38].

In this study, we examine the whole-body microbiomes of *I. scapularis* ticks from Broome County, located in the Southern Tier region of New York State. We analyzed the ticks’ whole-body microbiomes in order to gain a comprehensive overview of the microbial diversity present across all organs in *I. scapularis* ticks. We utilized a combination of 16S rRNA gene sequencing and mass spectrometry-based proteomics, with a subset of ticks screened by both methods. By doing so, we aim to provide the first report of the microbial compositions of female, male, and nymphal *I. scapularis* ticks from an unstudied region of New York. Furthermore, we examine possible associations between certain bacterial taxa and TBPs, as suggested by previous studies. Lastly, we evaluate the comparative ability of genetic and proteomic approaches in identifying the microbial compositions of *I. scapularis* ticks.

## 2. Materials and Methods

### 2.1. Tick Collection

*Ixodes scapularis* ticks were collected by dragging white corduroy fabric (1 m^2^) along bushes in trails in Chenango Valley State Park (42.21° N, −75.83° W) and Aqua-Terra State Park (42.03° N, −75.94° W) located in Broome County, New York. Latched ticks were carefully picked from the fabric using tweezers and were immediately placed in 1.5 mL polypropylene Eppendorf tubes containing 0.5 mL of 75% ethanol. The collected ticks were stored at −20 °C in 75% ethanol until processing (DNA or protein extraction).

### 2.2. Tick Processing for Genomic Microbiome Screening

Prior to DNA extraction, the ticks were pulverized using Qiagen TissueLyser LT and steel beads (Qiagen, Alameda, CA, USA). A single 5 mm bead was used for adult ticks, and three or four 3.2 mm beads were used for nymphal ticks. The tubes containing ticks and steel beads were agitated at 50 Hz for 2 min, or until the tick was thoroughly pulverized. DNA extraction was performed using the DNeasy Blood and Tissue Kit (Qiagen, Hilden, Germany). Pulverized ticks were incubated at 56 °C with 200 μL Buffer ATL and 20 μL Proteinase K for 18 to 24 h. The rest of the extraction process was conducted according to the manufacturer’s protocol. The quality and quantity of DNA in the extracted samples were assessed using a NanoDrop One spectrophotometer (Thermo Scientific, Waltham, MA, USA).

The DNA samples along with two negative DNA extraction controls (blanks) were processed for targeted 16S sequencing by a commercial provider (Microbiome Sequencing Services, Zymo Research, Irvine, CA, USA) using the Quick-16S™ Primer Set V3–V4 and NGS Library Prep Kit (Zymo Research, Irvine, CA, USA). This primer set contains two forward (341f: CCTACGGGDGGCWGCAG and CCTAYGGGGYGCWGCAG) and one reverse (806r: GACTACNVGGGTMTCTAATCC) primer. DNA libraries were cleaned up with Select-a-Size DNA Clean & Concentrator™ (Zymo Research, Irvine, CA, USA). Quantification was performed using a Qubit 2.0 fluorometer and the Invitrogen Qubit 1X dsDNA High-Sensitivity Assay (Thermo Fisher Scientific, Waltham, WA, USA). Libraries were pooled in equimolar amounts. A positive control (ZymoBIOMICS Microbial Community Standard) and a blank library preparation control were included. Sequencing was performed on the Illumina NextSeq 2000™ platform with the P1 600-Cycle Reagent Kit (Illumina, CA, USA). Unique amplicon sequences were inferred from the raw reads using the Dada2 pipeline [39]. The Dada2 pipeline was also used to remove chimeric sequences and potential sequencing errors. Taxonomy assignment of amplicon sequence variants (ASVs) was performed using Uclust from Qiime v.1.9.1. The analyses of within-sample (alpha) diversity and between-sample (beta) diversity of the microbial communities were performed with Qiime v.1.9.1 [40].

Data visualization was conducted in Rstudio v.4.4.1 [41] using the R package ‘ggplot2’ v.3.5.1 [42]. Principal component analysis (PCoA) was performed using the R package ‘vegan’ v.2.6.8 [43]. The co-occurrence of each pair of bacterial species was calculated using the ‘cooccur’ package v.1.3 [44]. Species with a relative abundance over 0.1% were assigned a value of “1”, and species with an abundance below 0.1% were assigned a value of “0”. This package uses presence–absence data points and calculates the probability that one site (an individual tick) contains both species 1 and 2, and whether they occur more or less frequently than expected. Positive or negative co-occurrence can be determined, given as “p_gt” and “p_lt”. These values are *p*-values to identify whether co-occurring species are significantly different.

### 2.3. Tick Processing for Proteomic Screening

To extract the total proteins from ticks, the excess ethanol was removed from each tube and the ticks were washed twice with ethanol, then once with PBS buffer (0.1 M, pH 7.4) to remove any outside contaminants. Each tick was then placed on absorbent towel paper, the legs and the gnathosoma or “head” were removed using a razor blade, and the remaining body was split horizontally into two pieces using the razor blade and transferred to a new polypropylene Eppendorf tube containing 50 μL of ice-cold RIPA buffer plus 1× protease inhibitor cocktail (Halt Protease Inhibitor Cocktail, Thermo Fisher Scientific). To extract the total proteins, handheld pellet pestles were used for 30 s while keeping the tubes on ice. The tubes were then vigorously vortexed for a few seconds and centrifuged at 16,000 rpm for 5 min. The supernatants were transferred into new 1.5 mL Eppendorf tubes, which were then placed on ice. The protein concentration in each sample was assessed using the bicinchoninic acid (BCA) assay (Thermo Fisher Scientific, Waltham, MA, USA). Aliquots containing 20 μg of proteins were taken from each sample, mixed with 1 μL of 200 mM DTT, and incubated for 15 min at 70 °C. Then, 5 μL of lithium dodecyl sulfate (LDS) sample buffer was added and the sample was vortexed and placed on a heating block at 95 °C for 5 min. The samples were then loaded onto 4–12% Bis-Tris polyacrylamide gel for electrophoresis (20 to 200 kDa range) and separation was performed at 150 V until the blue line ran to the bottom of the gel. The gels were subsequently rinsed with water and subjected to staining for a duration of 4 h with G250 Coomassie stain (Bio-Rad). The gel lanes containing the fractionated proteins were sliced into 2 to 3 mm sections using a razor blade, and each section was then transferred to a clean Eppendorf tube, where cysteine residues underwent reduction and alkylation before being processed for in-gel digestion according to a standardized protocol for Gel-LC–MS/MS [45]. The resulting peptides from each sample were analyzed using LC-MS/MS in data-dependent acquisition mode, and the raw mass spectra files were then collected and processed for protein identification, as previously described [36].

## 3. Results

### 3.1. Composition of Ixodes scapularis Whole-Body Microbiomes 

**Figure 1:**
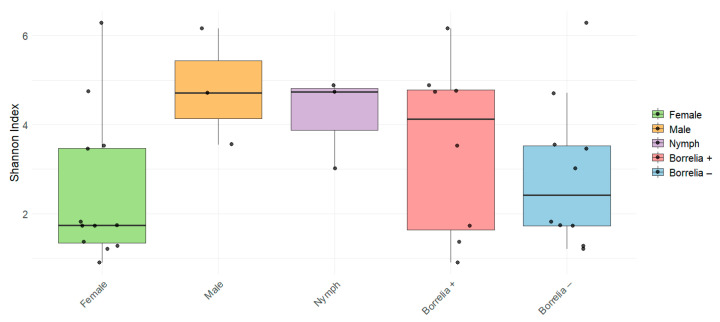
Boxplot of alpha diversity measured with the Shannon diversity index showing differences among tick groups.

We identified the microbial composition of 18 *I. scapularis* ticks (12 females, 3 males, and 3 nymphs) collected in Broome County, NY, using DNA extracted from the ticks’ whole bodies. The tick samples yielded an average of 833,650 raw reads, with a minimum of 602,710 reads per tick. After quality and size filtration, there were 326,664 reads per tick on average, with a minimum of 242,408 reads. These reads represented 114 to 347 unique 16S rRNA gene sequences per tick (mean = 191.72) (Table S1). Alpha diversity, as a measure of diversity within a sample, was calculated using the Shannon index. While male ticks exhibited a higher diversity than female ticks (Figure 1), this difference was not statistically significant (*p* = 0.07). A comparison based on *B. burgdorferi* infection also did not show a significant difference between *Borreliella*-positive (n = 8) and *Borreliella*-negative (n = 10) ticks; however, *Borreliella*-positive ticks had a larger range of diversity compared to *Borreliella*-free ticks.

**Figure 2:**
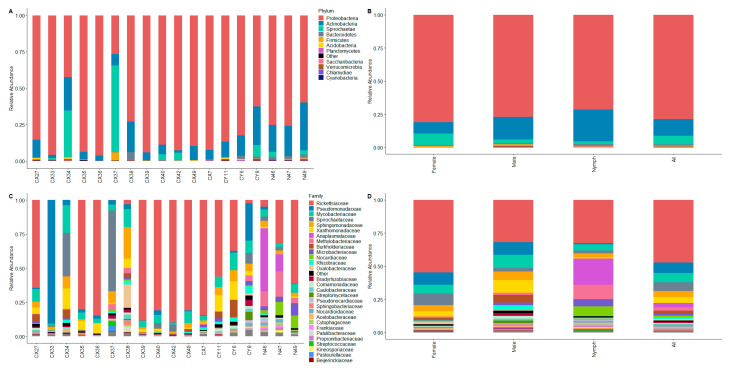
Microbial composition analysis of tick samples. (**A**) Relative abundance at the phylum level for 18 individual ticks. (**B**) Relative abundance at the phylum level for female, male, and nymph ticks grouped together. (**C**) Relative abundance at the family level for 18 individual ticks. (**D**) Relative abundance at the family level for female, male, and nymph ticks grouped together.

The most abundant bacterial phyla in the total sample of 18 ticks were Proteobacteria (78.65%), Actinobacteria (12.64%), and Spirochaetes (6.42%), followed by Bacteroidetes (0.90%) and Firmicutes (0.44%) (Figure 2A). One notable difference in the relative abundances of bacterial phyla between male, female, and nymphal ticks (Figure 2B) was a much lower abundance of Actinobacteria in female ticks (8.69%) compared to males (16.95%) and nymphs (24.12%). Instead, female ticks harbored Spirochaetes at a rate (8.48%) several times higher than that of male (2.66%) and nymphal ticks (1.97%). Spirochaetes were particularly abundant in the case of two female ticks (CX34 and CX37), comprising over 31% and 59% of their entire microbial communities.

The overabundance of the phylum Proteobacteria was mainly due to the high prevalence of the family Rickettsiaceae, accounting for an average of 47.29% of the amplicon sequence variants (ASVs) across the ticks. The other proteobacteria families that were abundant in our sample were Pseudomonadaceae (7.93%), Sphingomonadaceae (4.71%), Xanthomonadaceae (4.35%), and Anaplasmataceae (3.32%). Within the phylum Actinobacteria, Mycobacteriaceae was the most common family, accounting for an average of 6.90% of the bacterial communities across the sample. The phylum Spirochaetaceae comprised a single family, Spirochaetaceae (6.42%), which, along with Anaplasmataceae, represented the two tick-borne pathogen species detected in our sample (see next section). Together, these seven families accounted for about 90% of the total microbial population in our sample of 18 ticks. All other bacterial families had mean relative abundances below 3% (Figure 2C). The differences in the composition of the microbiomes between male, female, and nymphal ticks (Figure 2D) were more apparent on the family level compared to the phylum level. Most notably, Rickettsiaceae contributed to more than half of the microbiome in female ticks, compared to under a third of the total ASVs among male ticks and nymphs. The male ticks were distinguished from female ticks and nymphs by higher prevalences of Xanthomonadaceae, Burkholderiaceae, and Rhizobiaceae. In nymphs, on the other hand, neither of these three families reached 1% of the total microbiome. Conversely, the microbial communities in the whole bodies of nymphs contained relatively high loads of Methylobacteriaceae (10.98%), Nocardiaceae (7.18%), and Microbacteriaceae (5.11%), all of which were rare (relative abundance <1%) in adult ticks.

Out of the 60 most common genera accounting for 96.01% of the ASVs in our sample, a total of 42 genera had relative abundances of over 1% in at least one of the 18 ticks included in this study (Table S2). Two of these genera, *Pseudomonas* and *Bradyrhizobium*, also appeared in one of the extraction controls we analyzed along with our tick samples. Our analysis of the ASVs identified in the extraction controls supported an absence of cross contamination (See Supplementary File S1).

**Figure 3:**
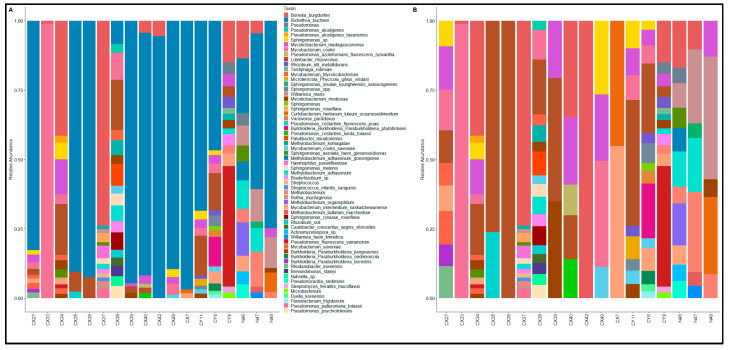
Microbial composition of 18 ticks at the species level. (**A**) Relative abundance of bacterial species across all 18 individual ticks. (**B**) Relative abundance of bacterial species across all 18 ticks after excluding *Rickettsia buchneri* to highlight the diversity of other species.

Identification of the microbiomes at the species level confirmed the dominant presence of the *I. scapularis* endosymbiont *Rickettsia buchneri* (Table S3). Present in 14 of the 18 ticks (77.77%), in relative abundances as high as 89.8%, *R. buchneri* was by far the most prevalent bacterial species (Figure 3). The two tick-borne pathogens detected in our sample, *B. burgdorferi* and *A. phagocytophilum*, were the second and fifth most abundant species, with average relative abundances of 6.42% and 3.29%, respectively. A *Pseudomonas* species detected in only two ticks was ranked as the third most abundant species (5.06%) due to it comprising over 88% of the microbial community in a single tick. The next two most abundant species were detected in the majority of the ticks: *Luteibacter rhizovicinus* (average abundance = 3.74%) comprised over 1% of the microbiome in 55.5% of ticks and *Mycolicibacterium madagascariense* (average abundance = 2.08%) was detected in all ticks, with relative abundances surpassing 1% in 61.1% of ticks.

The rest of the bacterial species presenting with relative abundances of over 1% in more than one tick belonged to the following genera: *Mycobacterium*, *Sphingomonas*, *Methylobacterium*, *Williamsia*, *Rhizobium*, *Paraburkholderia*, *Curtobacterium*, and *Bradyrhizobium*. All of these genera have been frequently reported in *I. scapularis* ticks and often compose the “core microbiome” of *I. scapularis* [28,46,47]. One notable observation was the high abundance of *Williamsia maris* in the tested nymphal ticks (average = 5.84%). This otherwise environmental actinomycete has been previously reported in *I. scapularis* [48,49].

#### 3.1.1. Beta Diversity

**Figure 4:**
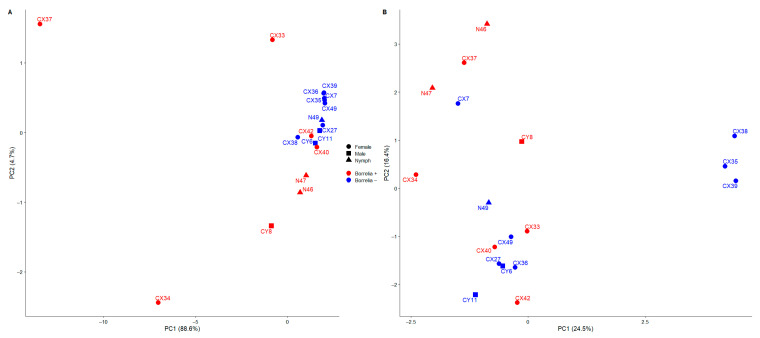
Principal coordinate analysis (PCoA) using (**A**) weighted UniFrac distances and (**B**) unweighted UniFrac distances measuring *Borreliella* positive (red) and negative (blue) samples.

We used principal coordinate analysis (PCoA) to visualize the weighted and unweighted UniFrac distances of bacterial communities according to tick life stage and the presence of *Borreliella*. The PCoA plot of the weighted UniFrac distances did not reveal a distinct clustering of ticks according to sex and life stage. When considering the *Borreliella* infectivity of ticks, this plot revealed a grouping of *Borreliella*-negative ticks regardless of sex and life stage (Figure 4A). It should be noted that two *Borreliella*-positive female ticks were also present in this cluster. Three other *Borreliella*-positive female ticks were the outliers in this plot. The PCoA plot of the bacterial communities according to the unweighted UniFrac distances did not show a separation of ticks by sex, life stage, or *Borreliella* infectivity (Figure 4B).

#### 3.1.2. Detection of Endosymbiont *Rickettsia* in Male *Ixodes scapularis* Ticks

In our study, *Rickettsia buchneri* was identified as the most abundant bacterial species in female *I. scapularis* ticks. In female ticks, *R. buchneri* was detected with an average relative abundance of 54.76% and comprised over 80% of the microbiome in 7 of the 14 female ticks. The endosymbiont *Rickettsia* was present in all three nymphs, with an average abundance of 32.71%. Interestingly, *R. buchneri* was detected in two of the three male ticks, with an average relative abundance of 31.15%. We conducted a co-occurrence test [44] to identify pairs of species for which the co-occurrence was significantly different than that expected by chance. A significant negative co-occurrence was detected for *R. buchneri* and *Pseudomonas fluorescens-yamanorum* (*p* = 0.02451). We did not identify a significant positive co-occurrence between *R. buchneri* and other species present in the microbiomes (Table S4).

#### 3.1.3. Tick-Borne Pathogens and *Ixodes scapularis* Microbiome

As anticipated, Lyme disease-causing *B. burgdorferi* was the most prevalent human pathogen (44.44%) detected in our sample. The relative abundance of *B. burgdorferi* in ticks ranged widely, from 1% to over 59% (Table S3). *Borreliella burgdorferi* had a positive co-occurrence with two *Methylobacterium* species (*p*-values = 0.02288 and 0.00654) and one *Actinomycetospora* species (*p* = 0.02288) (Table S4).

*A. phagocytophilum* was the other human pathogen present in the sample. While *A. phagocytophilum* was detected only in two nymphal ticks, our recent study showed no significant difference in *A. phagocytophilum* infectivity between nymphs and adults, with a prevalence of 7.31% [50]. Therefore, we attributed the absence of *A. phagocytophilum* in our 15 adult ticks to the small sample size. In the case of the two *A. phagocytophilum*-positive nymphal ticks, the anaplasmosis bacteria was highly abundant (12.73% and 46.46%), making it the second most abundant species after *R. buchneri* (Table S3). *Anaplasma phagocytophilum* had a negative co-occurrence with *Luteibacter rhizovicinus* (*p* = 0.01961). Otherwise, no significant co-occurrence was detected with other species.

### 3.2. Proteome Profiling of Single Ticks and Microorganism Identification

An additional, separate set of 36 ticks (22 females, 14 males) were processed for proteome profiling, as described in the Methods section. On average, 3077 proteins were identified in each tick, of which 1.2–2% belonged to various microorganisms. Table 1 shows the prevalence of the microorganisms of interest in ticks along with the list of the specific proteins representing each microorganism. Human pathogens were detected in female ticks only. The specificity of the proteins was verified manually through Uniprot BLAST using the identified peptide sequences. More than 80% of the female ticks carried *R. buchneri*, as identified by 12 unique proteins. The majority of these proteins were outer membrane proteins. Other intracellular proteins were also mapped to *R. buchneri*; however, they were excluded due to their sequences being homologous with those of other bacterial species. *Borreliella burgdorferi* was detected only in 3 of the 22 analyzed female ticks. This pathogen was identified by three unique proteins, including outer surface protein A (OspA), a flagellar filament 41 kDa core protein, and the integral outer membrane protein P66. Finally, 4 of the 22 female ticks carried the human parasite *Babesia microti*, which was identified by an uncharacterized protein according to the Uniprot knowledge database.

## 4. Discussion

### 4.1. Endosymbiont Rickettsia

The microbial composition of *I. scapularis* ticks collected in Broome County, New York, was generally consistent with previous reports from the Northeastern USA and Southeastern Canada. As with previous studies [34,51,52,53], *R. buchneri*, an endosymbiont of *I. scapularis*, was the most abundant bacterial species, comprising over half of the bacterial communities in female ticks. Tick endosymbionts are intracellular microorganisms that play crucial roles in their hosts’ biology, including nutrient provision, antimicrobial defense, and the modulation of the tick’s metabolic activities [54,55]. *Rickettsia buchneri* is known to synthesize essential nutrients like folate, which is important for tick development and reproduction [27]. In addition, *R. buchneri* may exert antimicrobial effects through its antibiotic clusters, contributing to the tick’s ability to resist infection by competing with pathogenic bacteria [30]. Recently, *R. buchneri* has been shown to regulate tick autophagy, which benefits its intracellular infection and replication [31].

*R. buchneri* is predominantly found in the ovaries and the developing oocytes of female ticks and is transmitted transovarially to offspring. However, limited studies have indicated the presence of *R. buchneri* in female salivary glands and male ticks [56,57]. In our study, we detected *R. buchneri* in two of three male ticks, with relative abundances of 37.21% and 56.23%, which were higher than previously reported values for male ticks [34,47,51,52,53]. Although the sample size for male and nymphal ticks in our study was small, the observed prevalence is noteworthy and supports the role of *R. buchneri* as a primary symbiotic bacterium throughout the tick life cycle. Our results revealed a 1.76-fold higher abundance of *R. buchneri* in females compared to males, which was comparable to the 2.2-fold difference reported for fed female and male ticks [57]. The higher abundance in female ticks could be due to the higher nutritional and reproductive demands of females, which might require greater symbiont abundance.

It should be noted that while the most abundant *R. buchneri* sequence in our data was 100% identical to the *R. buchneri* reference sequence [58], it was also identical to the reference sequence of *R. monacensis*, a closely related *Rickettsia* species isolated from *I. ricinus* ticks in Europe [59]. These two species are highly similar genetically, with their 16S genes being identical at the V3—V4 region. Nevertheless, we used the *R. buchneri* designation based on the findings of previous *I. scapularis* studies confirming the presence of this species in North America instead of the European *R. monacensis*.

As for interactions between the endosymbiont *R. buchneri* and other members of the *I. scapularis* microbial community, we observed a significant, negative co-occurrence with *Pseudomonas fluorescens-yamanorum* (these species cannot be distinguished using 16S V3—V4 sequences, but *P. yamanorum* has been rarely reported from North America [60,61], making it far less likely than *P. fluorescens*). In addition, the absence of *R. buchneri* in the male tick was associated with higher relative abundances of other genera, including *Streptomyces*, *Methylobacterium*, and *Bradyrhizobium*. On the other hand, the *R. buchneri*-positive male ticks were distinguished by 6-fold higher relative abundances of *Luteibacter* species (11.67% and 12.01%) compared to the *R. buchneri*-negative male tick (2.07%).

### 4.2. Microbiome-TBPs Interactions

Previous studies have reported associations between *B. burgdorferi* and a number of other bacteria, including a negative co-occurrence with *R. buchneri* [62,63]. A negative correlation may hint at adversarial interactions, such as the production of antimicrobial agents (e.g., by antibiotic-producing *P. fluorescens*), as well as competition. In our sample, while *B. burgdorferi* and *R. buchneri* demonstrated a negative co-occurrence, the association was not significant (*p* = 0.06863). That said, in the case of the three male ticks, there was a perfect negative correlation between *B. burgdorferi* and *R. buchneri*, suggesting a potential competitive interaction or a role of *R. buchneri* in modulating pathogen presence in male *I. scapularis*. We did not observe a negative co-occurrence between *B. burgdorferi* and *Pseudomonas* species, as previously suggested [27,28,29]. Instead, we report positive co-occurrences of *B. burgdorferi* with three environment-associated species (*Methylobacterium* and *Actinomycetospora*). Similar associations with other environmental bacteria have been reported previously [27,28].

In the case of *A. phagocytophilum*, a significant negative co-occurrence was observed with *Luteibacter rhizovicinus*, an environmental bacteria commonly identified in *Ixodes* ticks. We did not observe associations between *A. phagocytophilum* and *Rickettsia* (negative) or *Pseudomonas* (positive), as previously suggested [64]. Considering the potential role of *A. phagocytophilum* in enhancing tolerance to freezing [26], the high abundance of this bacteria in two nymphal ticks (46.46% and 12.73%) of Broome County, New York, might reflect the enhanced colonization of *A. phagocytophilum* in ticks living in the cold environment.

### 4.3. “Core Microbiome” of I. scapularis

In addition to *R. buchneri*, the core microbiome of Broome County *I. scapularis* ticks contained many of the same genera reported from other *I. scapularis* populations (*Pseudomonas*, *Mycobacterium*, *Sphingomonas*, *Methylobacterium*, *Williamsia*, *Rhizobium*, *Luteibacter*, *Paraburkholderia*, *Curtobacterium*, and *Bradyrhizobium*). We also detected *Bacillus*, *Staphylococcus*, and *Streptococcus* species, although with lower incidence and abundance compared to previous studies [28,46,65]. *Luteibacter* is a known member of the microbial community of *I. scapularis* [28,48,66] as well as other *Ixodes* species [32,67,68,69] and *Dermacentor* [70] ticks. In addition to *L. rhizovicinus*, 0.13% of the total sequences in our sample were 99.06% identical to those of both *L. anthropi* and *L. yeojuensis*, two species with indistinguishable 16S V3—V4 sequences. These species also have been detected in a previous study of the *I. scapularis* microbiome that reported a positive association between the presence of this genus and *Borreliella*-positivity in ticks [48]. As for *M. madagascariense*, we were able to confirm the specificity of a 462 bp sequence that was 100% identical only to this species, with 13 additional sequences showing the highest identity to *M. madagascariense*, at 99.8%. A second *Mycolicibacterium* species, *M. rhodesiae* (average abundance = 0.72%), was also detected in all ticks, with its relative abundance exceeding 1% in five ticks. We did not find a record of these two species in previous *I. scapularis* studies. However, *M. madagascariense* has been identified in *I. ricinus* ticks collected in Denmark [71]. This species is known to be present in soil and plants [72,73] and has also been detected in connection to *Ophiocordyceps highlandensis*, a fungus that grows on larvae of Scarabaeoidea beetles [74].

At just under 8%, *Pseudomonas* was the second most abundant genus in our sample, although excluding the single female tick (CX33) with an extreme abundance of *Pseudomonas* would reduce that value to 2.90%. This was comparable to the average abundance of *Pseudomonas* (3.5%) reported from *I. scapularis* collected in Vermont [28]. On average, *Pseudomonas* spp. were more abundant in the male ticks (9.58%) compared to females (8.60%, 1.77% when excluding CX33) and nymphs (0.80%). While this is consistent with some previous studies of *I. scapularis* from the Northeastern USA that report a much higher abundance of *Pseudomonas* in males (23%) compared to females (>1%) [47], a study of female ticks from New York identified *Pseudomonas* as the most abundant genus in this group (38.1%) [51]. Nevertheless, these studies agree on the higher prevalence and abundance of *Pseudomonas* in male *I. scapularis* from the Northeastern USA compared to the Southern USA (0.1–2.7% in Oklahoma and 1% in Texas) [47,51]. Other important observations were the high variability in *Pseudomonas* abundance within male, female, and nymphal groups and the negative co-occurrence of *Pseudomonas fluorescens-yamanorum* with *R. buchneri*. This was consistent with previous observations that, when *R. buchneri* is not the main component of the *I. scapularis* microbial community, *Pseudomonas* is likely to be the most abundant bacteria [29].

When analyzing microbial communities on a species level, the limitations of V3–V4 sequencing should be noted. Checking the sequences assigned as “*Pseudomonas palleroniana-tolaasii*” using BLAST (blastn), we were not able to verify their specific assignment to either of these two species. In fact, the assigned sequences were also 100% identical to a few members of the *Pseudomonas fluorescens* group, as well as some environmental *Pseudomonas* species previously reported from ticks [67]. Due to the 16S genes of all these species being identical in the V3–V4 region, a species-level assignment was impossible in this case. A similar observation was made in the case of *Methylobacterium* species identified in this study. This is a reminder of the limitations of partial 16S rRNA analysis in identifying bacterial species within certain taxa [75,76]. In such cases, the species-level assignment is somewhat speculative, and therefore, a genus-level analysis may be more informative. That said, a majority of the tentatively assigned *Pseudomonas* species in our sample belonged to the *P. fluorescens* group. *P. fluorescens* has been previously reported in *I. scapularis* ticks from New York and Vermont with relative abundances of 8% and <1%, respectively [28,51]. In both studies, non-fluorescens *Pseudomonas* species (*P. lurida* in NY and *P. putida* in VA) had a several-fold higher abundance than *P. fluorescens*. We detected *P. lurida* and *P. putida* in our sample in low abundances (<1%).

Overall, many members of the *I. scapularis* core microbiome were environment-associated bacteria largely reported in soil, water, and plant samples [48,49,65]. As discussed elsewhere [66], it is likely that the geographic and local variations in *I. scapularis* microbiomes reflect, to some extent, the differences in environmental bacteria present at the tick collection sites. In addition to regional differences, within the Northeastern USA, the microbial composition of *I. scapularis* seems to be determined by the specific location of sampling rather than sex [77].

As detailed in our review of the extraction controls (Supplementary File S1), we did not detect signs of DNA contamination in our samples. The bacterial taxa commonly known to be of human origin (members of skin and oral microbiome), e.g., *Staphylococcus*, *Streptococcus*, *Propionibacterium*, *Corynebacterium*, *Acinetobacter*, and *Cutibacterium*, were virtually absent in the microbiome results. The only exception was a female tick (bur) that contained *Haemophilus* and *Streptococcus* (3.60%). *Haemophilus* and *Streptococcus* species have been previously reported in *I. scapularis* ticks [49,53]; however, they are likely of a human origin.

### 4.4. The Proteome Signature of Tick-Borne Pathogens in Ixodes scapularis Ticks

In agreement with the 16S rRNA analysis, *R. buchneri* was by far the most abundant and frequently identified bacteria of interest in the proteomes of female ticks. Although the proteomic technique was less sensitive than 16S rRNA gene sequencing, it provided better specificity in the case of identified proteins. For example, the outer membrane protein B (ompB) was identified by 12 unique peptides that were mapped to the ompB protein belonging to the three *Rickettsia* species, including *R. buchneri* (*Rickettsia tamurae subsp buchneri*), *Rickettsia rickettsii* (strain Iowa), and *Rickettsia monacensis*. However, according to the Uniprot knowledge database, 2 out of the 12 identified peptides for ompB (AVDNVAYGIWTK, SSDENYKETSTTVANK) were unique to *R. buchneri*. OmpB is a large outer surface protein that is believed to play a role in the adhesion of *Rickettsia* species to host cells [78], and its methylation is believed to be implicated in the virulence of certain *Rickettsia* species, such as *R. prowazekii* and *R. typhi* [79]. However, *R. buchneri* is considered non-pathogenic to humans and other vertebrates, and it was shown to even inhibit the growth of other pathogenic *Rickettsia* in ticks [30]. Another *Rickettsia* protein, Porin domain-containing protein, was detected in ticks by 13 peptides, including two (NTVDSVSLATEYK and YTLLDGQYMR) that were unique to *R. buchneri* according to the Uniprot knowledge database. A few other proteins, such as adhesin and cell surface antigen Sca1, also generated tryptic peptide sequences unique to *R. buchneri*. Collectively, these data verified that the *Rickettsia* bacteria detected in *I. scapularis* female ticks collected from Broome County was the endosymbiont *R. buchneri*. Although *R. buchneri* was identified in male ticks in our 16S rRNA analysis, proteomic analysis failed to detect this species in male ticks, probably due to their inherent low abundance in male ticks. Additionally, potential transcriptional or translational regulation may limit the protein expression of *R. buchneri* in males, even when its genomic presence is detected.

Lyme disease-causing *B. burgdorferi* was identified by three specific proteins (OspA, Fla, and P66), which were detected in only 14% of the collected female ticks from Broome County in 2022. Interestingly, 18% of the female ticks gathered from Broome County in 2022 tested positive for *Babesia microti*, a protozoan parasite responsible for babesiosis, which presents with flu-like symptoms and anemia. The babesiosis condition is increasingly prevalent in New York State [80,81].

## 5. Conclusions

The application of 16S rRNA gene sequencing along with proteomics facilitated the precise and sensitive identification of tick-borne pathogens in individual ticks. Overall, our analysis of ticks collected in Broome County, New York, further supports the diversity of microbial communities present in *I. scapularis* ticks of the Northeastern USA. The observed prevalence and abundance of *R. buchneri* in male *I. scapularis* ticks, demonstrated using 16S rRNA gene sequencing, a technique that is more sensitive than proteomics, suggest a broader impact of this endosymbiont on the biology of male ticks than previously recognized. Our future studies will aim to confirm these findings with larger sample sizes and an analysis of specific tick organs (e.g., midgut and salivary glands) instead of the whole body. Given that *R. buchneri* is primarily associated with female ticks, investigating how this endosymbiont may influence male physiology, stress tolerance, and reproductive fitness can inform innovative strategies for tick and tick-borne disease control.

The presence of tick-borne pathogens in high proportions in a limited number of ticks indicates that certain ticks may serve as significant reservoirs for these pathogens in New York. This can have impacts on tick-borne disease transmission, as ticks with higher pathogen loads may be more likely to transmit diseases to hosts. Therefore, conducting spatial and temporal screening of ticks in high-risk locations is a highly valuable public health measure, as it can enhance awareness regarding the true risks associated with tick-borne diseases in the area. Tick screening can also assist healthcare providers in delivering personalized treatment based on the specific causative agents of diseases. Furthermore, proteomics offers a new avenue for diagnosis of tick-borne pathogens in clinical settings.

## Figures and Tables

**Table 1:** Prevalences of pathogens and *R. buchneri* in female and male *I. scapularis* ticks collected in Broome County, NY, according to the proteomic analysis. Among the 22 female ticks, 15 (68.18%) carried *R. buchneri*, 3 (13.64%) carried *B. burgdorferi*, and 4 (18.18%) carried the parasite *B. microti*. No pathogens were detected in the 14 male ticks.

Microorganism	Identified Protein	Number of Positive Ticks	
		Female (n = 22)	Male (n = 14)
*Rickettsia* *buchneri*	Adhesin (A0A8E1C0T9)	10	0
	Adhesin A0A8E1C0I9)	8	0
	Outer membrane protein B (A0A8E0WKQ0)	12	0
	Cell surface antigen Sca1 (A0A8E0WLZ4)	4	0
	Porin domain-containing protein (A0A8E0WLM2)	6	0
	Peptidoglycan-associated lipoprotein (A0A8E1C013)	3	0
	Parvulin-like PPIase (A0A8E0WMR4)	4	0
	Spore protein SP21 (A0A8E1BZB2)	7	0
	Hsp20/alpha crystallin family protein (A0A8E0WKN8)	10	0
	Biotin synthase (A0A8E1BZ92)	2	0
	Transposase (A0A8E1BZI6)	3	0
	Putative adhesin (A0A0B7J122)	5	0
Total number of ticks with *R. buchneri* proteins		15	0
*Borreliella* *burgdorferi*	Outer surface protein A (P0CL66)	2	0
	Flagellar filament 41 kDa core protein (P11089)	3	0
	Integral outer membrane protein P66 (A0A0H3C4D7)	1	0
Total number of ticks with *B. burgdorferi* proteins		3	0
*Babesia microti*	Uncharacterized protein (A0A1N6LY41) [KLVSDYCSLGR]	4	0
	Uncharacterized protein (A0A1N6LY97) [TFGNFSLFEK]	1	0
Total number of ticks with *B. microti* proteins		4	0

## Data Availability

The original contributions presented in this study are included in the article/Supplementary Material. Further inquiries can be directed to the corresponding author.

## Supplementary Materials

The following supporting information can be downloaded at: https://www.mdpi.com/article/10.3390/microorganisms13020258/s1, File S1: Analysis of DNA extraction controls; Table S1: Summary of 16S rRNA gene amplicon reads per tick; Table S2: Relative abundance (%) of 16S rRNA gene amplicon sequences per tick by genera. “CX” and “CY” indicate female and male ticks, respectively; Table S3: Relative abundance (frequencies) of the most common species per tick; Table S4: Results of the co-occurrence analysis of pairs of bacterial species present in the sample of 18 ticks. Only co-occurrent species with statistical significance (*p* < 0.05) are shown. References [82,83] are cited in the Supplementary Materials.

## Abbreviations

The following abbreviations are used in this manuscript:

TBD	Tick-borne Disease
TBP	Tick-borne Pathogen
Subsp	Subspecies
Spp.	Species
