# Supplementary material for: Genomic and Proteomic Analyses of Bacterial Communities of Ixodes scapularis Ticks from Broome County, New York
Source: Microorganisms. 2025 Jan 24;13(2):258. doi: 10.3390/microorganisms13020258 (PMC11857480; doi:10.3390/microorganisms13020258)
Supplement: Supplementary file 1 [file microorganisms-13-00258-s001.zip › Supplementary File S1.pdf]

Supplementary File S1

Article

Genomic and Proteomic Analysis of Bacterial Communities of *Ixodes scapularis* Ticks from Broome County, New York

Michel Shamoon-Pour, Emily H Canessa, John Macher, Amaan Fruitwala, Emma Draper, Benjamin Policriti, Matthew Chin, Matthew Nunez, Paul Puccio, Yuan Fang, Xin-Ru Wang and Yetrib Hathout

Analysis of DNA Extraction Controls

Out of a total of 60 genera detected in our samples, including two extraction controls, two genera (*Pseudomonas* and *Bradyrhizobium)* were also present in one of the extraction controls (EC1) we analyzed along with our tick samples. We reviewed the presence of *Pseudomonas* and *Bradyrhizobium* in the microbial compositions of ticks as a sign of possible contamination. In case of *Pseudomonas,* the relative abundance of this genus exceeds 4% of total microbiome in one female (CX33) and one male (CY8) tick. In fact, a staggering 90.57% of all ASVs detected for CX33 belong to *Pseudomonas*. The further breakdown of *Pseudomonas* ASVs detected for CX33 shows that almost all (>97%) of them belong to one species: *Pseudomonas palleroniana-tolaasii*. However, none of the *Pseudomonas* ASVs detected for the EC1 extraction control were specifically assigned to this species. Similarly, in case of CY8, a vast majority of *Pseudomonas* ASVs belong to a single species (*Pseudomonas fluorescens-yamanorum*) which was not detected in EC1, an observation that does not support cross contamination as a source of *Pseudomonas* in these ticks. As for *Bradyrhizobium,* which was the second common bacteria detected in EC1, small quantities of the same species was in fact detected in three ticks, including CY8 (2.13%) and CX33 (1.29%). Considering the fact that *Bradyrhizobium* is a soil bacteria previously reported in ticks in considerable quantities (Maldonado-Ruiz et al., 2021; Thapa et al., 2019; Ponnusamy et al., 2014), the *Bradyrhizobium* detected for ticks are likely endogenous, with the DNA extracted from ticks being the source of *Bradyrhizobium* ASVs found in EC1 (Table S3). As for the second extraction control (EC2), the three species detected in this control (*Escherichia*

coli, *Propionibacterium acnes*, *Granulicatella adiacens*) are typically known from human samples and as common causes of DNA contamination. These three species were virtually absent from all 18 ticks, supporting an absence of cross contamination.

References:

Thapa, S.; Zhang, Y.; Allen, M. S. Bacterial Microbiomes of Ixodes Scapularis Ticks Collected from Massachusetts and Texas, USA. *BMC Microbiol.* **2019**, *19* (1), 138. https://doi.org/10.1186/s12866-019-1514-7.

Maldonado-Ruiz, L. P.; Neupane, S.; Park, Y.; Zurek, L. The bacterial community of the lone star tick (Amblyomma americanum). *Parasites & Vectors* **2021**, *14*. https://doi.org/10.1186/s13071-020-04550-z.

Ponnusamy, L.; Gonzalez, A.; Van Treuren, W.; Weiss, S.; Parobek, C. M.; Juliano, J. J.; Knight, R.; Roe, R. M.; Apperson, C. S.; Meshnick, S. R. Diversity of rickettsiales in the microbiome of the lone star tick, Amblyomma Americanum. *Applied and Environmental Microbiology* **2014**, *80*, 354–359. https://doi.org/10.1128/aem.02987-13.
